# Prophylactic Dendritic Cell-Based Vaccines Efficiently Inhibit Metastases in Murine Metastatic Melanoma

**DOI:** 10.1371/journal.pone.0136911

**Published:** 2015-09-01

**Authors:** Oleg V. Markov, Nadezhda L. Mironova, Sergey V. Sennikov, Valentin V. Vlassov, Marina A. Zenkova

**Affiliations:** 1 Institute of Chemical Biology and Fundamental Medicine, Siberian Branch of Russian Academy of Sciences, Novosibirsk, Russia; 2 Federal State Budgetary Institution "Research Institute of Clinical Immunology", Siberian Branch of Russian Academy of Medical Sciences, Novosibirsk, Russia; INSERM, FRANCE

## Abstract

Recent data on the application of dendritic cells (DCs) as anti-tumor vaccines has shown their great potential in therapy and prophylaxis of cancer. Here we report on a comparison of two treatment schemes with DCs that display the models of prophylactic and therapeutic vaccination using three different experimental tumor models: namely, Krebs-2 adenocarcinoma (primary tumor), melanoma (B16, metastatic tumor without a primary node) and Lewis lung carcinoma (LLC, metastatic tumor with a primary node). Dendritic cells generated from bone marrow-derived DC precursors and loaded with lysate of tumor cells or transfected with the complexes of total tumor RNA with cationic liposomes were used for vaccination. Lipofectamine 2000 and liposomes consisting of helper lipid DOPE (1,2-dioleoyl-*sn*-glycero-3-phosphoethanolamine) and cationic lipid 2D3 (1,26-Bis(1,2-de-O-tetradecyl-*rac*-glycerol)-7,11,16,20-tetraazahexacosan tetrahydrocloride) were used for RNA transfection. It was shown that DCs loaded with tumor lysate were ineffective in contrast to tumor-derived RNA. Therapeutic vaccination with DCs loaded by lipoplexes RNA/Lipofectamine 2000 was the most efficient for treatment of non-metastatic Krebs-2, where a 1.9-fold tumor growth retardation was observed. Single prophylactic vaccination with DCs loaded by lipoplexes RNA/2D3 was the most efficient to treat highly aggressive metastatic tumors LLC and B16, where 4.7- and 10-fold suppression of the number of lung metastases was observed, respectively. Antimetastatic effect of single prophylactic DC vaccination in metastatic melanoma model was accompanied by the reductions in the levels of Th2-specific cytokines however the change of the levels of Th1/Th2/Th17 master regulators was not found. Failure of double prophylactic vaccination is explained by Th17-response polarization associated with autoimmune and pro-inflammatory reactions. In the case of therapeutic DC vaccine the polarization of Th1-response was found nevertheless the antimetastatic effect was less effective in comparison with prophylactic DC vaccine.

## Introduction

One novel therapeutic approach to curing cancer is to enlist the body’s natural defence mechanisms and recruit key contributors to the immune response. Anti-tumor vaccines based on dendritic cells (DCs) are promising for tumor prophylaxis and therapy due to their ability to generate T cell mediated anti-tumor responses, prevent systemic metastasis, provide long-lasting anti-tumor effects and, therefore, prevent tumor recurrence [1,2]. DCs are the most applicable tools for activation of anti-tumor immune responses because DCs are antigen-presenting cells that have the ability to capture, process and present tumor-associated antigens (TAA) to naive lymphocytes, subsequently triggering and controlling antigen-specific anti-tumor immune responses [3].

The most common approach to anti-tumor therapy is the use of DCs as a therapeutic (post-exposure) vaccine [4]. To prepare a therapeutic vaccine, precursors of DCs are isolated from the peripheral blood mononuclear cell population and/or bone marrow cells of patients and immature DCs are generated with appropriate growth factors. Immature DCs are pulsed with tumour antigens in the form of peptides [5], proteins [6], tumour cell lysates [7], apoptotic [8, 9] or live [10] tumour cells, as well as viral vectors [11–13] and nucleic acids (NA) encoding TAA [14] *ex vivo* and re-administered to the recipient, where DCs migrate into peripheral lymph nodes and spleen and initiate the activation of anti-tumor T and B cell immune responses. This technique is well established and widely used [15–18]; however, it is still “necessary to know the tumor is present in order to act”. Currently DCs are being broadly investigating as therapeutic cancer vaccines in various clinical trials against melanoma [19], small cell lung cancer [20], glioma [21], glioblastoma [22], hepatocellular carcinoma [23], etc. In 2010 the first USA FDA approval ever of a therapeutic DC-based cancer vaccine, Sipuleucel-T, to treat advanced castration-resistant prostate cancer was granted [24].

The use of DCs as a prophylactic (pre-exposure) anti-tumor vaccine is still studied *in vivo*. These vaccines may be of interest for prophylaxis of people with a genetic predisposition to cancer where a high risk of malignant neoplasm development exists. For example, women carrying highly penetrant mutations in the *BRCA1* and *BRCA2* genes have an elevated risk of developing breast (*BRCA1/2* carriers, 36–90%) and ovarian cancer (*BRCA1* carriers, 24–59%; *BRCA2* carriers, 8–35%) [25,26].

Pre-exposure vaccines may also be of interest to patients with elevated levels of tumor markers in the blood that indicate a risk of tumor development. For example, they will be of interest for patients with high concentrations of prostate-specific antigen (PSA), which indicates a significant risk of prostate cancer development in men [27]. Other markers such as CA125 (MUC126) and AFP (alpha fetoprotein) are not cancer specific; nevertheless, their high levels in serum are used for early diagnosis of ovarian cancer and hepatocellular carcinoma, respectively [28]. Actually, the use of prophylactic vaccines is necessary in situation where a tumor has not yet been discovered but nevertheless there is a high probability of one developing.

Usually in experiments *in vivo* using prophylactic treatment schemes DCs are loaded by the same antigens as in the case of therapeutic settings [29–32] except that allogeneic tumor material are used instead of autologous tumor material. Thus when prophylactic DC vaccines are exploited together with the use of TAA-encoding constructs for DC loading material from allogeneic cell lines of tumors is the only possible antigen source mimicking autologous tumor material in the absence of tumor.

There have been a few attempts to use DCs as pre-exposure vaccines *in vivo* and encouraging results have been obtained for inhibition of primary tumor growth [30–36] but in few cases metastases [37–39]. Herein we performed extensive research where we compared pre-exposure and post-exposure vaccination with DCs using three different experimental tumor models, that have strong relevance to human tumors—namely, Krebs-2 adenocarcinoma (primary tumor), corresponds to human adenocarcinoma; B16 melanoma (metastatic tumor without a primary node), corresponds to widespread metastatic human melanoma, and Lewis lung carcinoma (LLC) (metastatic tumor with a primary node), corresponds to anaplastic human carcinoma. Dendritic cells of bone marrow origin loaded with lysate or total RNA from tumor cells were used for vaccination. To deliver tumor RNA into DCs commercially available Lipofectamine 2000 (hereinafter LF) or cationic liposomes 2D3-DOPE (hereinafter 2D3) consisting of helper lipid DOPE (1,2-dioleoyl-*sn*-glycero-3-phosphoethanolamine) and cationic lipid 2D3 (1,26-Bis(1,2-de-O-tetradecyl-*rac*-glycerol)-7,11,16,20-tetraazahexacosan tetrahydrocloride) were used. 2D3-DOPE liposomes were chosen due to its ability to deliver RNA into murine DC progenitors and immature DCs with high efficacy and to stimulate their maturation [40]. We demonstrated the benefit of prophylactic RNA-loaded DC vaccines to treat highly aggressive metastatic tumors (melanoma B16 and Lewis lung carcinoma), whereas therapeutic RNA-loaded DC vaccines were more efficient against non-metastatic tumor (Krebs-2).

## Materials and Methods

### Cell lines and tumor models

B16, the C57Bl/6J-derived melanoma line, was purchased from the Institute of Cytology (Russian Academy of Sciences, St. Petersburg, Russia). B16 cells were maintained in DMEM supplemented with 10% fetal bovine serum (FBS), 100 U/ml penicillin, 100 μg/ml streptomycin, and 0.25 μg/ml amphotericin in a humidified atmosphere containing 5% CO_2_ at 37°C, and were passaged regularly to maintain exponential growth. The LLC and Krebs-2 tumor strains were obtained from the vivarium at the Institute of Cytology and Genetics, Siberian Branch, Russian Academy of Sciences (SB RAS), Novosibirsk, Russia.

Three tumor models differing in the presence of primary tumor nodes and metastasis were used in the work, namely a solid form of Krebs-2 adenocarcinoma (Krebs-2) without metastasis, a solid form of Lewis lung carcinoma (LLC) with both a tumor node and metastasis and a metastatic model of B16 melanoma (B16) without a primary tumor node. Two schemes of mouse treatment with DC vaccines were used, featuring prophylactic and therapeutic immunizations.

### Mice

Male 10–14 week-old C57Bl/6J (hereinafter, C57Bl/6) mice were obtained from the animal breeding facility within the Institute of Cytology and Genetics SB RAS (Novosibirsk, Russia) and not maintained in SPF conditions. Mice were housed in plastic cages (6–10 animals per cage) under normal daylight conditions. Water and food were provided ad libitum. All animal procedures were carried out in strict accordance with the approved protocol and recommendations for proper use and care of laboratory animals [European Communities Council Directive 86/609/CEE]. The protocols were approved by the Committee on the Ethics of Animal Experiments of the Administration of Siberian Branch of Russian Academy of Sciences (Protocol Number: 19-04-2013) and all efforts were made to minimize suffering.

At the start of the experiments animals weighed (mean±S.E.M.) 20.2±1.5 grams. To perform both Krebs-2 and LLC studies 6–8 animals per group were used. To perform melanoma B16 studies 5–11 animals per group were used. The number of animals in the group was chosen that was sufficient to perform statistical analysis and to get significant data.

### Generation of immature DCs

DC progenitors were obtained from the bone marrow of C57Bl/6 mice by density gradient centrifugation through Histopaque-1083 medium (Sigma, USA). Immature DCs were generated by culturing of DC progenitors in IMDM supplemented with 10% FBS and 1% antibiotic-antimycotic solution (10 mg/ml streptomycin, 10 000 IU/ml penicillin, and 25 μg/ml amphotericin; ICN, Germany) in the presence of 20 ng/ml GM-CSF (Invitrogen, USA) and 50 ng/ml IL-4 (Invitrogen, USA) at 37°С in a humidified atmosphere containing 5% CO_2_ for 6 days.

### Preparation of Krebs-2 cell lysate and total RNA from tumor cells

Krebs-2 tumor cells were isolated from ascites by density gradient centrifugation through Histopaque-1083 medium and resuspended at a density of 5×10^6^ cell/ml in IMDM without serum and antibiotics. Cells were frozen at −70°С for 20 min and then thawed at room temperature. The freeze/thaw cycles were repeated four times. The lysate was centrifuged at 300g for 1 min and the supernatant was passed through a 0.22 μm nitrocellulose filter. The protein concentration was determined using a Total Protein Kit, Micro-Lowry, Peterson`s Modification (Sigma, USA) in accordance with the manufacturer’s recommendations. The lysate was stored at −20°С. The isolation of total RNA from tumor cells was performed using TRIzol Reagent (Invitrogen, USA) according to the manufacturer’s protocol.

### Loading of DCs with antigens

After 6 days of culture DCs were collected and incubated with Krebs-2 lysate (120 μg of protein/10^6^ DCs) at 37°С for 3 h or transfected with tumor derived total RNA in complexes with Lipofectamine 2000 (Invitrogen, USA) according to the manufacturer’s protocol or with 2D3-DOPE cationic liposomes as previously described [40].

Phenotype of mature DCs after loading with RNA in complexes with liposomes was analysed by flow cytometry using monoclonal antibodies against the DC markers CD80, CD83, CD86 and MHC II conjugated with FITC or PE (Invitrogen, USA). Cell surface staining was performed following standard flow cytometry procedures. Samples were analyzed on a FC 500 flow cytometer (Beckman Coulter, USA).

### Vaccine composition and schedules for DC vaccination of mice

#### DC vaccines

Type of DC vaccines is indicated as S/T/A where S—Scheme of the treatment 1 (prophylactic) or 2 (therapeutic), T—Transfectant, A—Antigen source, or S-I/T/A where S—Scheme of the treatment 1 (prophylactic) or 2 (therapeutic), I—Immunization number, T—Transfectant, A—Antigen source. For the Krebs-2 adenocarcinoma (Krebs-2) model, DC vaccines consisted of DCs loaded with total RNA from tumor cells in the presence of Lipofectamine 2000 (1/LF/RNA, 2/LF/RNA) or tumor lysate (1/lysate, 2/lysate) or intact DCs treated with Lipofectamine 2000 (1/LF, 2/LF). For Lewis lung carcinoma (LLC), DC vaccines consisted of DCs transfected with total tumor-derived RNA in the presence of Lipofectamine 2000 (1/LF/RNA, 2/LF/RNA) or 2D3-DOPE cationic liposomes (1/2D3/RNA, 2/2D3/RNA). For the B16 melanoma DC vaccine, we used DCs transfected with total RNA isolated from B16 melanoma cells in the presence of cationic liposomes 2D3-DOPE (1-1/2D3/RNA, 1-2/2D3/RNA, 2-1/2D3/RNA, 2-2/2D3/RNA). Viability of prepared DCs was tested just before mice immunization by staining with trypan blue and was about 60–75%. All DC vaccines were administered intravenously (i.v.) at a concentration of 10^5^ cells/mouse in 0.2 ml of PBS. Each time freshly prepared DCs were used for DC vaccine.

#### Scheme 1 (prophylactic, pre-exposure vaccine)

Krebs-2: Healthy mice were randomly divided into three groups (n = 6) and received i.v. DC vaccines: (1)– 1/LF; (2)– 1/LF/RNA; (3)– 1/lysate. On day 7 after DC vaccination tumors were induced in mice by intramuscular injection of Krebs-2 cells (10^5^ cells/mouse) into the femur muscle of right hindfoot. LLC: Healthy mice were randomly divided into two groups (n = 6–8) and received i.v. DC vaccines: (1)– 1/LF/RNA; (2)– 1/2D3/RNA. On day 7 after DC vaccination tumors were induced in mice by intramuscular injection of LLC cells (6×10^5^ cells/mouse) into the femur muscle of right hindfoot. B16: Healthy mice were randomly divided into two groups (n = 5–6) and received i.v. DC vaccines: (1)– 1-1/2D3/RNA on day 7 before tumor transplantation (single DC vaccination); (2)– 1-2/2D3/RNA on day 14 and 7 before tumor transplantation (double DC vaccination). B16 was induced by transplantation of B16 cells (10^5^ cells/mouse) into lateral tail vein. In prophylactic scheme as controls we used controls of therapeutic scheme corresponding to tumor type. No adverse events were observed.

#### Scheme 2 (therapeutic, post-exposure vaccine)

Tumors were induced in mice by intramuscular injection of Krebs-2 cells (10^5^ cells/mouse) or LLC cells (6×10^5^ cells/mouse) into the femur muscle of right hindfoot or intravenous inoculation of B16 cells (10^5^ cells/mouse) into lateral tail vein. Krebs-2: On day 4 after tumor transplantation mice were randomly divided into four groups (n = 6) and received i.v. DC vaccines: (1, w/t)–saline buffer; (2)– 2/LF; (3)– 2/LF/RNA; (4)– 2/lysate. LLC: On day 4 after tumor transplantation mice were randomly divided into three groups (n = 6–8) and received i.v. DC vaccines: (1, w/t)–saline buffer; (2)– 2/LF/RNA; (3)– 2/2D3/RNA. B16: On day 4 after tumor transplantation mice were randomly divided into three groups (n = 5–11) and received i.v. DC vaccines: (1, w/t)–saline buffer; (2)– 2-1/2D3/RNA on day 4 after tumor transplantation (single DC vaccination); (3)– 2-2/2D3/RNA on day 4 and re-vaccination on day 11 (double DC vaccination). No adverse events were observed.

Mice were sacrificed under light ether anesthesia on days 19–20, 20 or 15 in the Krebs-2, LLC and B16 tumor models, respectively. Tumor volumes in the experimental animals were measured with callipers every 2–3 days during the experiments. Tumor volume was calculated as (length×width×height)/2. After the end of the experiment the numbers of lung metastases were counted for the metastasized LLC and B16 tumors. Both prophylactic and therapeutic experiments were repeated, and data were pooled.

### Evaluation of CD4+/CD8+ T cell ratio in spleens of experimental animals (Krebs-2 model)

Spleens were obtained from experimental animals on days -7, 0 and 19 for prophylactic DC vaccination (Scheme 1) or days 0, 5 and 11 in the case of therapeutic DC vaccination (Scheme 2). Spleens were homogenized and erythrocytes were lysed with RBC lysis buffer (0.15 M NH4Cl, 10 mM NaHCO3, 0.1 mM Na2EDTA). Then spleen cells were isolated by density gradient centrifugation through Histopaque-1083 medium (Sigma, USA). Spleen cells were washed with PBS and stained in Stain Buffer (BD Pharmingen, USA) containing 2% Fetal Bovine Serum with anti-mouse CD4 PE (Invitrogen, USA) and anti-mouse CD8 FITC (Invitrogen, USA) mAbs for 1 h on ice. After double washing with PBS, the cells were fixed with 4% formaldehyde and analysed with a Cytomics FC 500 (Beckman Coulter, USA).

The percentage of CD4 PE-positive cells in analyzed samples was calculated using equation CD4-PE^+^(%) = CD4-PE^+^_sample_ (%)−CD4-PE^+^_control_ (%), where CD4-PE^+^_sample_ (%)—a percentage of fluorescence-positive cells in the analyzed sample, CD4-PE^+^_control_ (%)—a percentage of fluorescence-positive cells in the negative control. Cells incubated in the absence of CD4-PE were used as a negative control: in these samples the percentage of fluorescence-positive cells did not exceed the experimental error (1–3%). The percentage of CD8 FITC-positive cells in analyzed samples was calculated using the same equation. Gating strategy is presented in S1 Fig.

### Measurement of cytokine levels in blood serum

Blood samples were collected from all groups of animals treated with DC vaccines in the B16 model on day 15 after tumor transplantation: group 1, the control, received saline buffer intravenously; groups 2 and 3 received single and double prophylactic DC vaccines intravenously (10^5^ cells/mouse), respectively; groups 4 and 5 received single and double therapeutic DC vaccines intravenously (10^5^ cells/mouse), respectively. Blood serum was prepared by clot formation at 37°C for 30 min and at 4°C overnight followed by clot discard and serum centrifugation (4000 rpm, 4°C, 20 min). Serum samples were stored at −70°C until analysis. The levels of IL-1β, IL-2, IL-4, IL-5, IL-6, IL-10, IL-12, GM-CSF, IFN-γ and TNF-α in the blood serum of the mice were measured using the Mouse Cytokine 10-Plex Panel (Invitrogen, USA) according to the manufacturer’s protocol.

### Real time RT-PCR

The expression of RORg, Foxp3, GATA3 and Tbet were detected by EVA-Green-based real-time RT-PCR. RNA was prepared from 10^7^ spleen cells obtained from the groups of mice: healthy mice; non-treated mice with metastatic melanoma, mice with metastatic melanoma treated with DC vaccines 1-1/2D3/RNA, 1-2/2D3/RNA, 2-1/2D3/RNA and 2-2/2D3/RNA. RNA isolation was performed using TRIzol Reagent (Invitrogen, USA) according to the manufacturer’s protocol. RNA (1 μg) was reverse transcribed to cDNA and subjected to real time PCR. The PCR mixture (20 μl), consists of 5 μl of cDNA (10^−2^ dilution), 10 μl of EVA-Green-containing the PCR master mix (Vector-Best, Russia) and 0.4 μmol of each primer. The following primers were used: RORg sense 5’-GGAGCTCTGCCAGAATGACC-3’, RORg antisense 5’-CAAGGCTCGAAACAGCTCCAC-3’; Foxp3 sense 5’-CAGCTGCCTACAGTGCCCCTA-3’, Foxp3 antisense 5’-CATTTGCCAGCAGTGGGTAG-3’; GATA3 sense 5’-TACTTGCGTTTTTCGCAGGA-3’; GATA3 antisense 5’-GATCTGTCGCTTTCGGGCCT-3’; Tbet sense 5’-CGGTACCAGAGCGGCAAGT-3’, Tbet antisense 5’-CATGCTGCCTTCTGCCTTTC-3’; β-actin sense 5’-TCCACCA CCACAGCTGAGAGG-3’, β-actin antisense 5’-CAGCTTCTCTTTGATGTCACG-3’ [41, 42]. The cycling condition were 95°C for 15 s, 75°C for 1 min, 60°C for 30 s, for 40 cycles, followed by a melting point determination or dissociation curves. The expression level of each gene is indicated by the number of cycles needed for the cDNA amplification to reach a threshold. The amount of DNA is calculated from the number of cycles by using standard curves and the results are normalized to β-actin.

### Statistics

The experimenter measuring and calculating the primary animal data (tumor size, metastases number) was blinded. After unblinding the animal data were statistically processed using one-way ANOVA. Post-hoc testing was completed using Fisher’s least significant differences (LSD). p <0.05 was considered to be statistically significant. Statistical package STATISTICA version 10.0 has been used for analysis.

## Results

### Characteristics of DCs

DC vaccines were obtained from bone marrow-derived DC precursors which underwent differentiation in the presence of GM-CSF and IL-4 for 6 days and then were loaded with tumor associated antigens in the form of tumor cell lysate or total tumor-derived RNA complexed with commercially available Lipofectamine 2000 (here and after LF) or with 2D3-DOPE cationic liposomes (here and after 2D3) [40].

**Fig 1:**
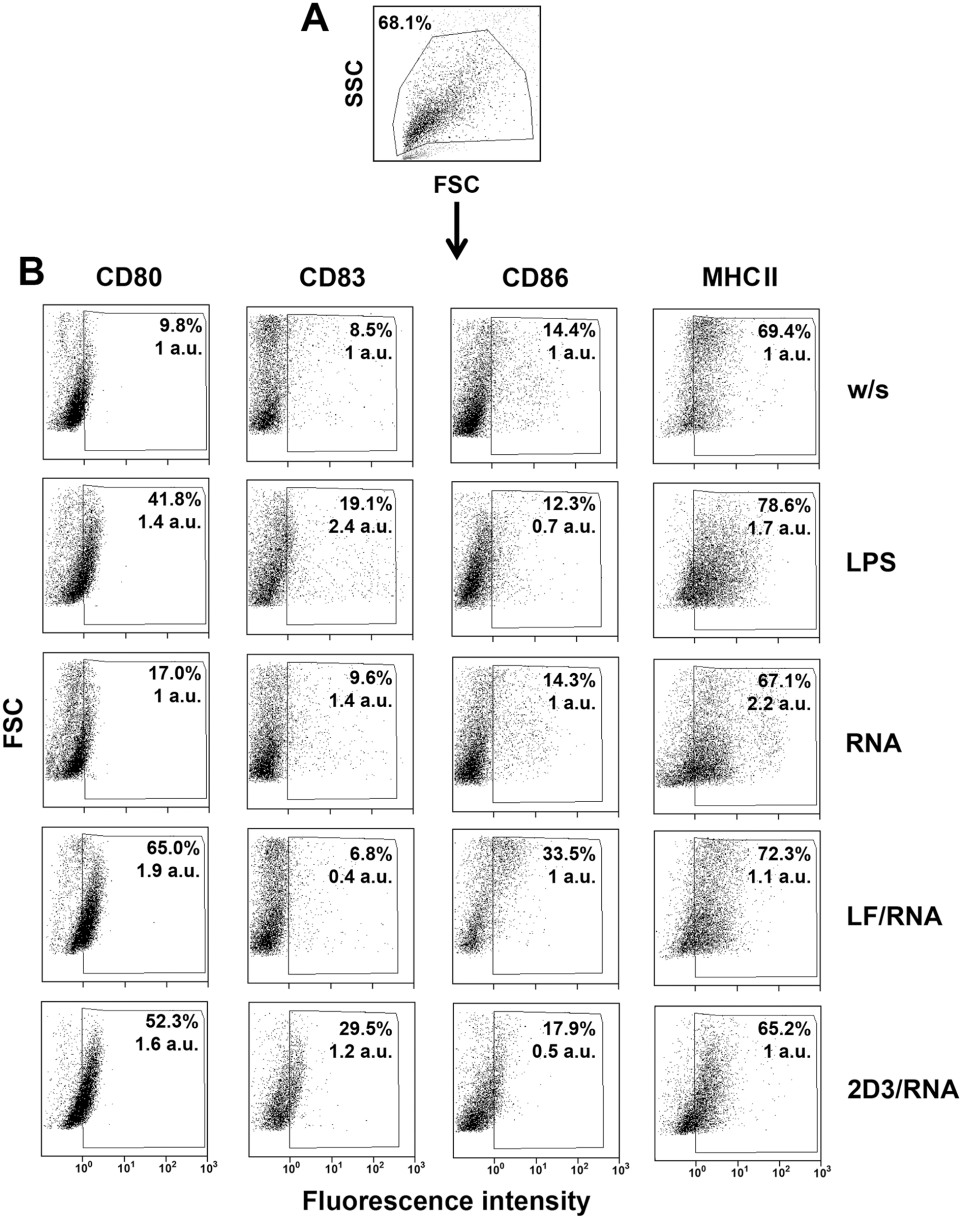
Activation status of bone-marrow-derived murine dendritic cells after transfection with lipoplexes LF/RNA and 2D3/RNA. A. Dead cells were excluded from DC population according to size and structure of cells. B. Surface expression of CD80, CD83, CD86 and MHC II by DCs after transfection with lipoplexes LF/RNA and 2D3/RNA. DCs without any stimulation (w/s) and treated with naked RNA isolated from B16 cells served as negative controls. DCs treated with LPS served as a positive control. DCs were stained with anti-CD80 (phycoerythrin), anti-CD83 (phycoerythrin), anti-CD86 (fluorescein isothiocyanote) and anti-MHC II (phycoerythrin), and were analyzed by flow cytometry. Ten thousand cells were counted for each sample. Data are presented as percent of fluorescent cells and mean fluorescence intensity of whole population of DCs normalized to w/s group (in arbitrary units, a.u.).

DC phenotype after loading was studied using mAb against maturation markers CD80, CD86, CD83 and MHC II (Fig 1). Non-loaded DCs (Fig 1B, w/s panel) expressed low amounts of maturation markers and their phenotype was close to immature or slightly mature (probably due to the influence of FBS in the culture medium). In the case of DCs treated with LPS an increase of expression level of three maturation markers CD80, CD83 and MHC II was observed (Fig 1B, compare w/s and LPS panels) both in terms of fluorescent cell number and mean fluorescence intensity (MFI). It is seen that DCs stimulated by lipoplexes of LF or 2D3-DOPE with RNA-B16 expressed some maturation markers and most probably corresponded to matured DCs. Stimulation of DCs with LF/RNA caused six-fold increase of CD80^+^ cells together with two-fold increase of MFI, two-fold increase of CD86^+^ cells and slight increase of MHC II^+^ cells as well as MFI in comparison with non-stimulated DCs (Fig 1B, compare w/s and LF/RNA panels). Stimulation of DCs with 2D3/RNA resulted in five-fold increase of CD80^+^ cells together with 1.6-fold increase of MFI and three-fold increase of CD83^+^ cells accompanied by slight increase of MFI in comparison with non-stimulated DCs (Fig 1B, compare w/s and 2D3/RNA panels).

### Comparison of anti-tumor activity of prophylactic and therapeutic DC vaccinations in the different tumor models

**Fig 2:**
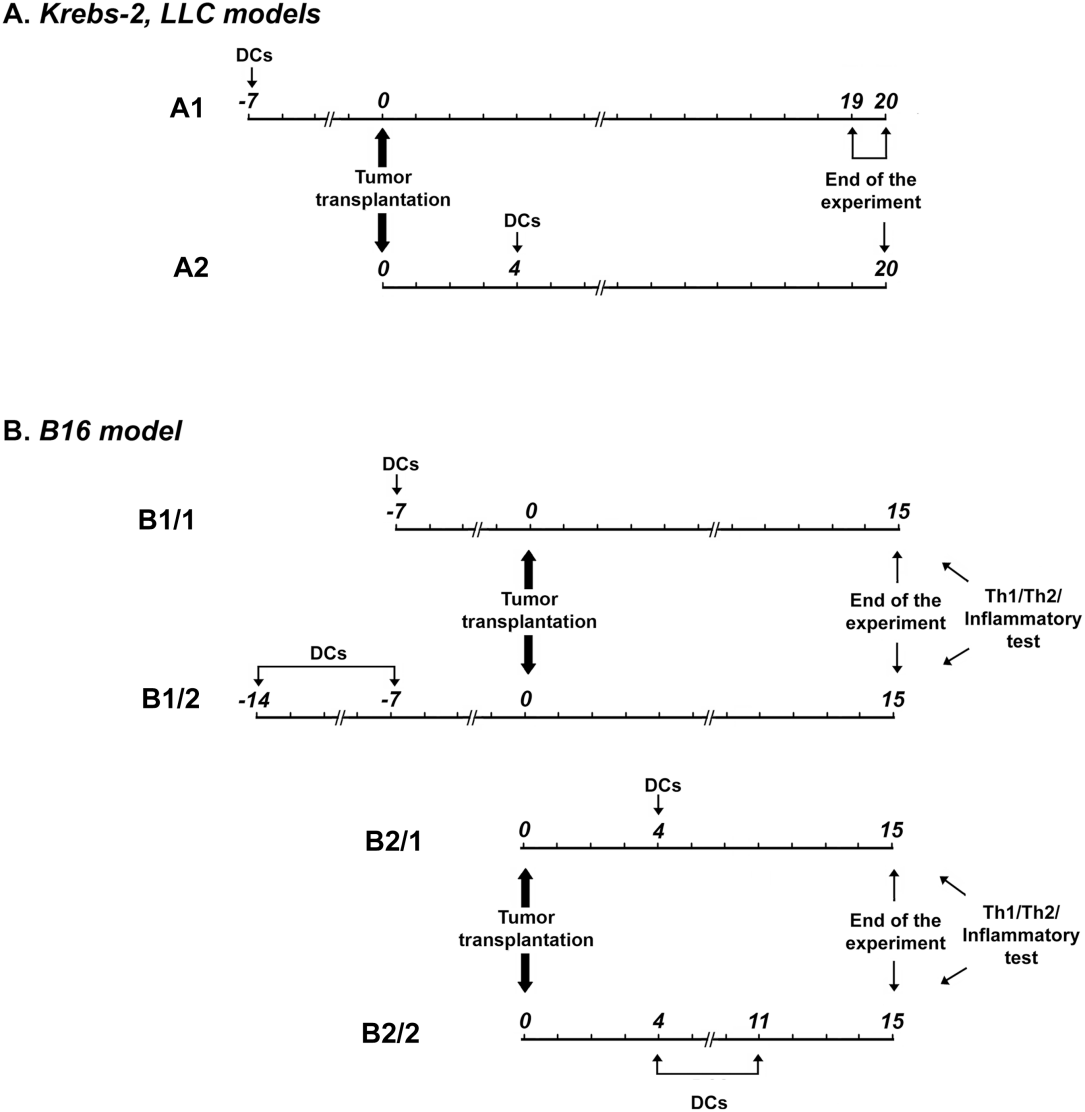
Experimental schedules of mouse treatments with DC vaccines. (A) Treatment of Krebs-2 and LLC tumors. Scheme 1 (prophylactic): Mice were injected with DC vaccines intravenously. A week later Krebs-2 or LLC cells were intramuscularly transplanted into mice. Mice with LLC and mice with Krebs-2 were sacrificed on days 19–20 and 20, respectively. Scheme 2 (therapeutic): Krebs-2 or LLC tumor cells were intramuscularly transplanted into the femur muscle of right hindfoot of mice, mice were treated with DC vaccines intravenously on day 4 and sacrificed on day 20. (B) Treatment of B16 tumors. Scheme 1 (prophylactic): Mice were intravenously injected with a DC vaccine once or twice with a one-week interval. A week after the last DC injection, B16 cells were inoculated intravenously into the mice. The mice were sacrificed on day 15. Scheme 2 (therapeutic): B16 cells were intravenously transplanted into mice, mice received one or two DC vaccines intravenously on day 4 or days 4 and 11, respectively. The mice were sacrificed on day 15.

The experimental schedules of DC immunization are presented in Fig 2. DC vaccines were i.v. injected into mice in all schemes of DC immunization. The i.v. route of administration was chosen because it was demonstrated that murine gp33-loaded DCs injected i.v. in the dosage that we used were as effective as DCs injected s.c. in Lewis lung carcinoma and melanoma B16 tumor models [14]. Furthermore, i.v. inoculation of DC vaccines could be more adapted for translation to clinical application in humans. As a source of antigen for preparation of DC vaccines we used tumor lysate or total tumor-derived RNA.

Fig 2A1, 2B1/1 and 2B1/2 illustrate prophylactic vaccination. In the Krebs-2 and LLC tumor models, mice were immunized once with a DC vaccine a week before tumor transplantation (Fig 2A1). In the B16 model one or two DC immunizations with a one week interval in between were used—initial immunization was performed two weeks before tumor transplantation, booster vaccination was performed a week before tumor transplantation (Fig 2B1/1 and 2B1/2). Fig 2A2, 2B2/1 and 2B2/2 illustrate therapeutic anti-tumor vaccination. Mice received DC vaccines on day 4 after tumor transplantation, and revaccination of mice in the B16 model was performed on day 11, a week after the first vaccination.

**Fig 3:**
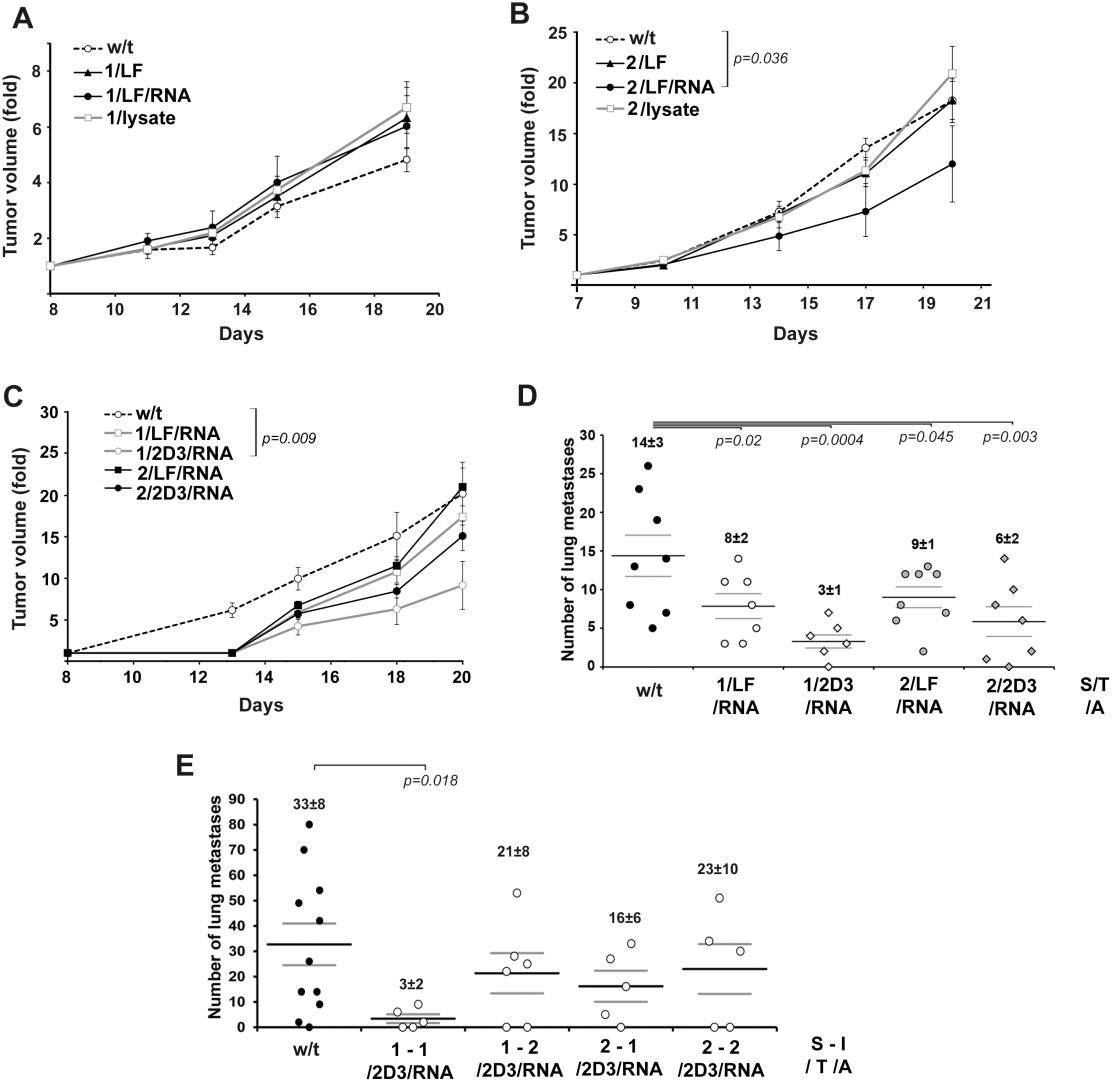
Anti-tumour and anti-metastatic effects of DC vaccination under prophylactic and therapeutic schemes. A and B. Krebs-2 adenocarcinoma growth retardation after treatment with DC vaccines. w/t—non-treated mice with Krebs-2 injected with saline buffer. C and D. LLC tumor growth retardation and suppression of metastasis after treatment with DC vaccines. For A-D type of DC vaccine is presented as S/T/A—Scheme/ Transfectant/ Antigen source. w/t—non-treated mice with LLC injected with saline buffer. E. Suppression of B16 melanoma metastasis after treatment with DC vaccines. For E type of DC vaccine is presented as S-I/T/A—Scheme—Immunization number/ Transfectant/ Antigen source. w/t—non-treated mice with metastatic melanoma injected with saline buffer. Data were statistically analysed using one-way ANOVA with post hoc Fisher test. Data are presented as mean±S.E.M. *p* value <0.05 was considered to be statistically significant. Scheme 1: Healthy mice received i.v. DC vaccines according to presented S/T/A type. On day 7 after DC vaccination tumors were induced in mice by intramuscular injection of Krebs-2 cells (10^5^ cells/mouse) or LLC cells (6×10^5^ cells/mouse) into the femur muscle of right hindfoot. In the case of B16 model healthy mice received i.v. Dc vaccines according to presented S-I/T/A type: on day 7 before tumor transplantation (S-I: 1–1) and on day 14 and 7 before tumor transplantation (S-I:1–2). B16 was induced by transplantation of B16 cells (10^5^ cells/mouse) into lateral tail vein. Scheme 2. Tumors were induced in mice by intramuscular injection of Krebs-2 cells (10^5^ cells/mouse) or LLC cells (6×10^5^ cells/mouse) into the femur muscle of right hindfoot or intravenous inoculation of B16 cells (10^5^ cells/mouse) into lateral tail vein. On day 4 after tumor transplantation mice received i.v. Dc vaccines according to presented S/T/A or S-I/T/A type.

The anti-tumor effects of prophylactic and therapeutic vaccinations are presented in Fig 3. DC vaccination with the prophylactic scheme did not affect tumor growth regardless of the antigen source (lysate or total RNA). Our data show that in the Krebs-2 model the highest anti-tumor activity was observed in the case of therapeutic vaccination (Fig 3B). Administration of the DC vaccine under Scheme 2 resulted in discernible inhibition of tumor growth: in the group of mice treated with 1/LF/RNA DC vaccine (abbreviation corresponds to Scheme of the treatment/ Transfectant/ Antigen source), tumor size was decreased 1.9-fold in comparison with the control group (*p* = 0.036, Fig 3B). Administration of 2/lysate under the therapeutic scheme did not lead to an apparent retardation of tumor growth in comparison with the control group and the group treated with 1/LF.

Since the DC vaccine based on DCs loaded with total tumor RNA exhibited greater efficiency in the Krebs-2 model in comparison with DCs loaded with tumor lysate, in experiments with the LLC model total RNA from an LLC tumor was used as a source of tumor associated antigens for DC loading. Two types of transfectants—Lipofectamine 2000 and cationic liposomes 2D3-DOPE having enhanced delivery potential [40]—were used for RNA delivery into DCs. Fig 3C and 3D shows the dynamic of LLC tumor growth and the number of lung metastases after the prophylactic and therapeutic vaccinations. It should be noted that treatment with neither the prophylactic nor therapeutic DC vaccines 1/LF/RNA and 2/LF/RNA caused significant LLC growth retardation. In the group of animals treated under prophylactic Scheme 1 with 1/2D3/RNA, a 2-fold retardation of tumor growth was observed (*p* = 0.009, Fig 3C). At the same time the highest level of metastasis inhibition was observed in this experimental group: treatment of mice with the prophylactic vaccine 1/2D3/RNA resulted in a 4.7-fold reduction in the number of lung metastases in comparison with the control group (*p* = 0.0004, Fig 3D). Therapeutic vaccination with a similar DC vaccine (2/2D3/RNA) resulted in a 2.3-fold suppression in the number of metastases (*p* = 0.003, Fig 3D). Both types of vaccination with 1/LF/RNA and 2/LF/RNA led to a 1.5-fold reduction in the number of lung metastases (*p* = 0.02 and *p* = 0.045, respectively, Fig 3D).

To achieve a highly efficient immune response in the B16 model, single and double (initial and booster) DC vaccinations were performed (Fig 2B). DCs transfected with RNA-B16/2D3 lipoplexes were used as DC vaccines. The single prophylactic vaccination 1-1/2D3/RNA was the most effective and caused a 10-fold decrease in the number of metastases (*p* = 0.018, Fig 3E). The second immunization under prophylactic scheme 1-2/2D3/RNA also caused the decrease of metastases number similar to therapeutic Scheme 2 and did not result in reinforcement of the anti-metastatic effect: the inhibition of metastasis was 1.5-fold and was comparable with the treatment efficiency of 2-1/2D3/RNA and 2-2/2D3/RNA under therapeutic Scheme 2 (1.5–2-fold inhibition) (Fig 3E). In both the prophylactic and therapeutic schemes, double immunizations with DC vaccines were less effective than a single one.

### Immunologic characteristics of prophylactic and therapeutic treatment of tumors with DC vaccines

**Fig 4:**
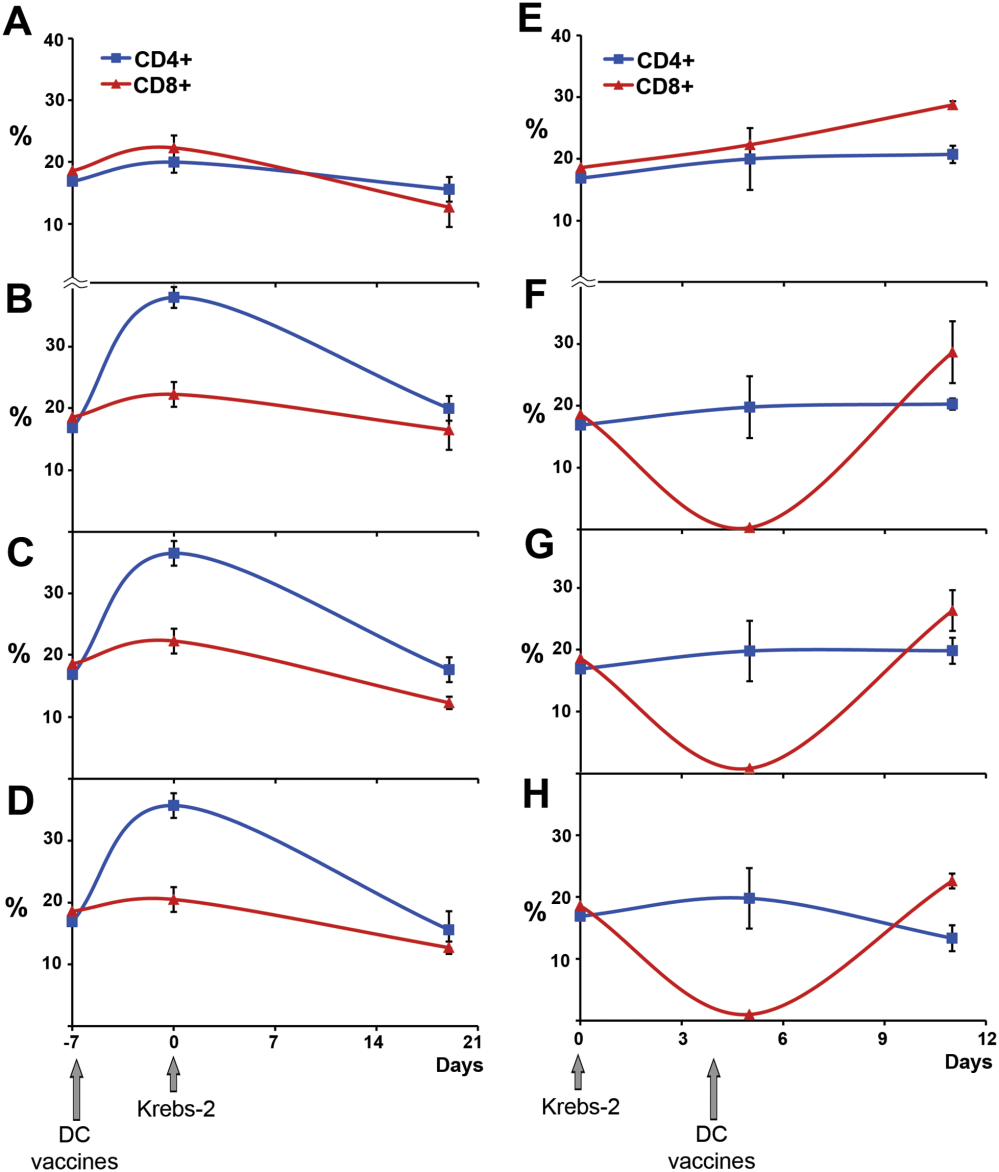
CD4+ and CD8+ cell content in spleens of animals with Krebs-2 treated with prophylactic and therapeutic DC vaccines. A. w/t, non-treated mice with Krebs-2 injected with saline buffer, time of tumor development 19 days. B, C and D. Mice received prophylactic DC vaccines 1/LF, 1/LF/RNA and 1/lysate, respectively. E. w/t, non-treated mice with Krebs-2 injected with saline buffer, time of tumor development 11 days. F, G and H. Mice received therapeutic DC vaccines 2/LF, 2/LF/RNA and 2/lysate, respectively. CD4+ and CD8+ content at the point -7 days was measured just before DC vaccination and corresponded to baseline. Type of DC vaccine is presented as S/T/A—Scheme of the treatment 1 or 2/ Transfectant/ Antigen source. Blue line indicates CD4+ cells, red line—CD8+ cells. Arrows indicate the day of DC vaccination and the day of tumor transplantation. Data are presented as mean±S.E.M. All experimental points were run in triplicate.

In the beginning we studied CD4^+^ and CD8^+^ cells content, reflecting the balance between T helper and T cytotoxic responses, in the spleens of mice with Krebs-2 after prophylactic or therapeutic DC vaccination (Fig 4). Gating strategy of flow cytometry is presented in S1 Fig. CD4^+^ and CD8^+^ cell content at the point -7 days (Fig 4A–4D) and point 0 days (Fig 4E–4H) was measured before DC vaccination and tumor transplantation thus correspond to baseline of healthy mice. Baseline CD4^+^ cell content was 18%, baseline CD8^+^– 19%.

In the control group of prophylactic scheme (non-treated animals with Krebs-2, Fig 4A) CD4^+^ content slightly varied during tumor development. Prophylactic DC vaccines 1/LF, 1/LF/RNA and 1/lysate caused a 2-fold increase in CD4^+^ cell content on day 7 after vaccination independent of antigen source (Fig 4B, 4C and 4D). In these groups by the end of the experiment (day 19) the number of CD4^+^ cells decreased to the level of animals of control group and was in the range of 18–22%. CD8^+^ cell content slightly decreased from the baseline (19%) to 13–17% by the end of experiment in all groups including control (Fig 4A–4D). Thus, under prophylactic scheme the content of CD4+ cells varied while the content of CD8+ cells remained almost unaffected.

In control group of therapeutic scheme (non-treated animals with Krebs-2) CD4^+^ cell content slightly changed during tumor development from 17% (baseline) to 21% while CD8^+^ cell content significantly increased from 19% (baseline) to 29% by day 11 (Fig 4E). Therapeutic DC vaccines 2/LF and 2/LF/RNA did not affect CD4^+^ cell content during tumor development (Fig 4F and 4E). In the case of DC vaccine 2/lysate CD4^+^ cell content decreased up to 13% by the end of the experiment (Fig 4H). In contrast to CD4+ cells all therapeutic DC vaccines 2/LF, 2/LF/RNA and 2/lysate caused almost complete disappearance of CD8^+^ cells on day 1 after DC vaccination (Fig 4F, 4G and 4H). However, on day 7 after DC vaccination the number of CD8^+^ cells in the groups 2/LF and 2/LF/RNA returned to the level of the control (29%) or was somewhat lower (2/lysate, 22% CD8+ cells).

To evaluate the immune status of experimental animals with B16 melanoma after either prophylactic or therapeutic DC vaccination, three important components of the immune response were examined—T helper 1 (Th1), T helper 2 (Th2) and pro-inflammatory responses. Concentrations of specific cytokines were measured in the blood serum of experimental animals on day 15 of the experiment (see Fig 2). It turned out that DC vaccination under prophylactic scheme did not result in statistically significant differences in the content of cytokines specific for the Th1 response (see S2 Fig). In the case of therapeutic scheme the increase of IL-2 and IL-12 (p40/p70) levels was observed in the groups 2-1/2D3/RNA and 2-2/2D3/RNA, respectively.

**Fig 5:**
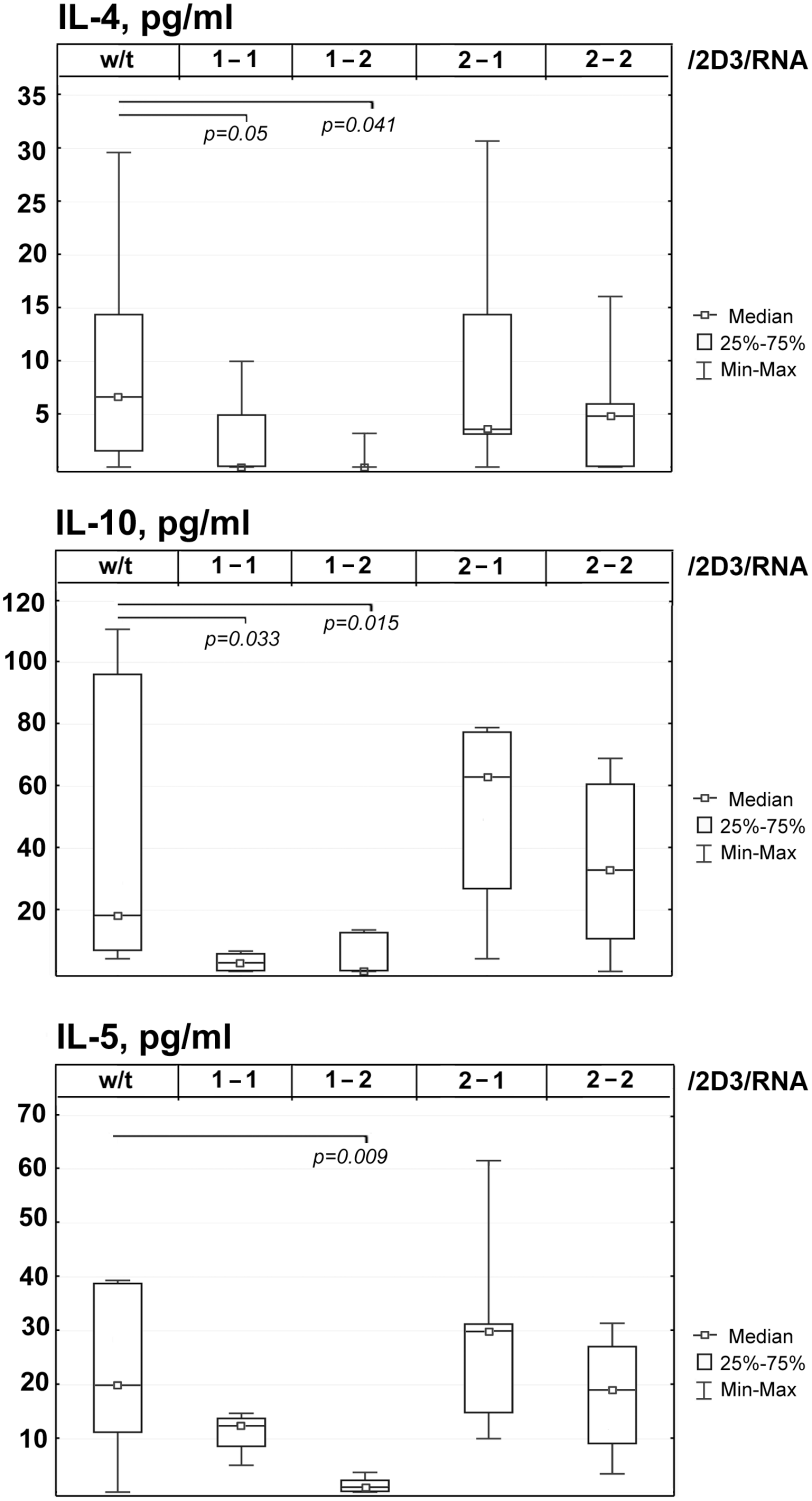
Box plots of Th2-specific cytokine content in the blood serum of mice with metastatic melanoma after prophylactic or therapeutic DC vaccination: (A) IL-4, (B) IL-10, (C) IL-5. Boxes represent 25^th^, 50^th^, and 75^th^ percentiles. Squares with line represent median. Whiskers represent minimum/maximum. w/t—non-treated mice with metastatic melanoma injected with saline buffer. Type of DC vaccine is indicated as S-I/T/A where S—scheme of the treatment 1 or 2, I—immunization number, T—transfectant, A—antigen source. Data were statistically analysed using one-way ANOVA with post hoc Fisher test. *p* value indicates a statistically reliable difference.

However, in the groups of mice treated with the prophylactic DC vaccine, reductions in levels of cytokines specific for the Th2 response were observed (Fig 5). We noted the disappearance of IL-4 (*p* = 0.05 and *p* = 0.041 for groups 1-1/2D3/RNA and 1-2/2D3/RNA, respectively, Fig 5A), a 6-fold decrease in IL-10 (*p* = 0.033 and *p* = 0.015 for groups 1-1/2D3/RNA and 1-2/2D3/RNA, respectively, Fig 5B). Also we observed 1.6-fold decrease in IL-5 for group 1-1/2D3/RNA (data are statistically insignificant, Fig 5C) and almost complete disappearance of IL-5 for group 1-2/2D3/RNA (*p* = 0.009, Fig 5C). Concentrations of T helper 2 specific cytokines in the blood serum of mice treated with therapeutic DC vaccines 2-1/2D3/RNA and 2-2/2D3/RNA did not differ in comparison with the control group.

Analysis of pro-inflammatory cytokines in the blood serum of treated mice revealed that prophylactic vaccine 1-1/2D3/RNA caused approximately 3-fold increase of TNF-α (*p* = 0.001, S3 Fig). Vaccine 2-1/2D3/RNA resulted in 1.3-fold increase of IL-1β level (*p* = 0.008, S3 Fig).

### Determination of master transcription factors Tbet, RORg, GATA3 and Foxp3

**Fig 6:**
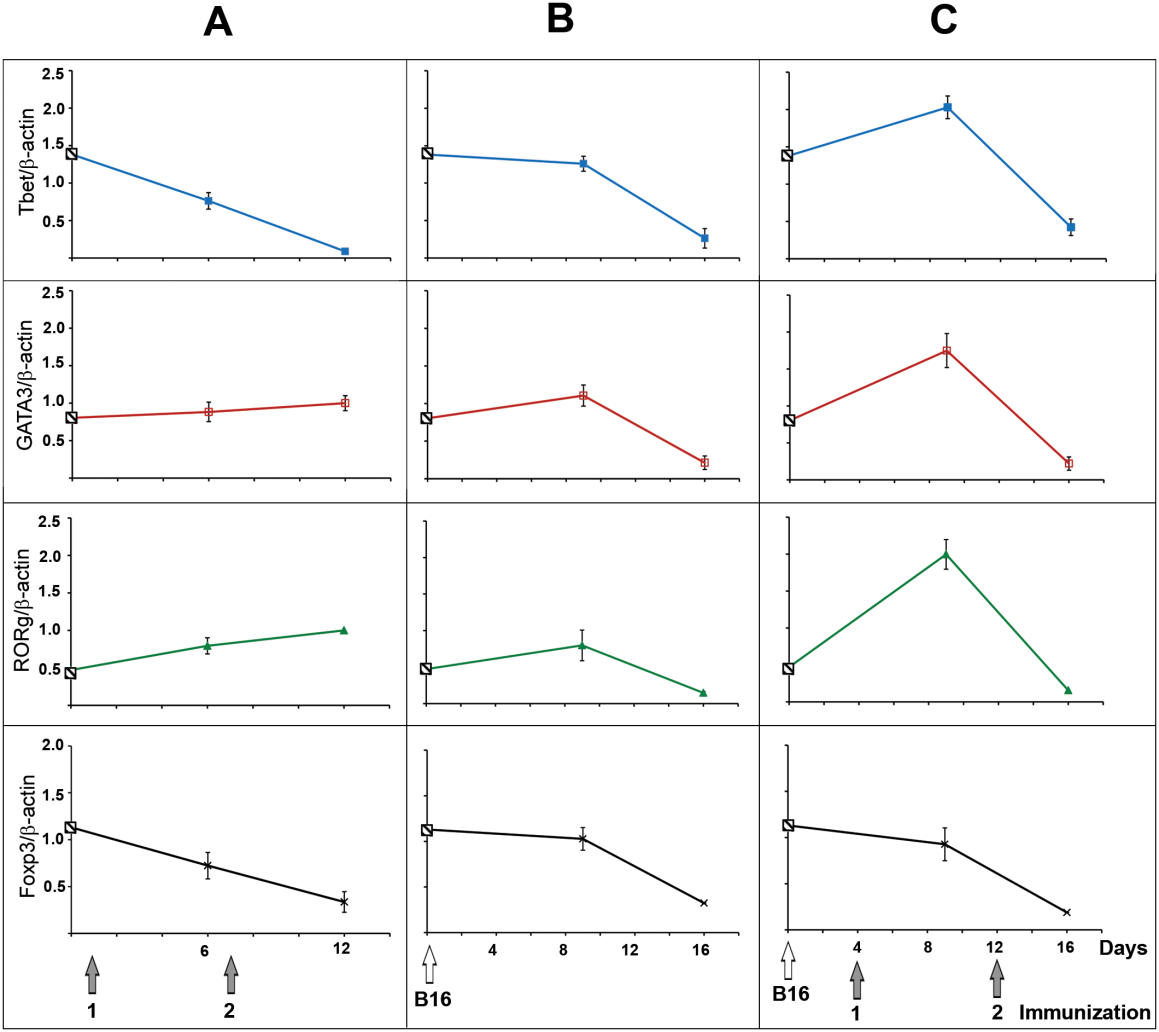
The expression level of Tbet, GATA3, RORg, and Foxp3 in spleen cells of mice after DC vaccination (qPCR data). A. Prophylactic treatment scheme: healthy mice receiving DC vaccines 1-1/2D3/RNA and 1-2/2D3/RNA (1 and 2 immunization, respectively). B. Mice with metastatic melanoma receiving saline buffer. C. Therapeutic treatment scheme: mice with metastatic melanoma receiving DC vaccines 2-1/2D3/RNA and 2-2/2D3/RNA (1 and 2 immunization, respectively). Crossed square on Y axis displays the level of gene expression in healthy intact mice (baseline). Type of DC vaccine is indicated as S-I/T/A where S—scheme of the treatment 1 or 2, I—immunization number, T—transfectant, A—antigen source. Expression of the genes in group without treatment was measured on days 9 and 16 of tumor development. Expression of the genes in prophylactic and therapeutic groups was measured on day 5 after each immunization. Data represent the mean ± SD of three experiments performed in triplicate. Data were statistically analysed using one-way ANOVA with post hoc Fisher test.

Since analysis the level of Th1- and Th2-specific cytokines in blood plasma of mice with metastatic melanoma after DC vaccination did not let to determine exactly the type of Th response we studied the expression of master transcription factors Tbet (Th1), GATA3 (Th2), RORg (Th17) and Foxp3 (Treg) by real time RT-PCR in spleen cells of mice received DC vaccines (Fig 6). It was shown that both prophylactic vaccines have no effect on the expression level of GATA3 (Fig 6A): the levels of this gene did not differ from baseline (healthy animals, point on Y axis, Fig 6A–6C) and w/t group (non-treated mice with metastatic melanoma). With the increase of immunization number we observed disappearance of Tbet (p = 0.0018) and 2-fold increase of RORg (p = 0.04). Also three-fold decrease of the expression level of Foxp3 in comparison with baseline was observed but it was statistically insignificant (Fig 6A).

First immunization with DC vaccine of mice with metastatic melanoma resulted in the increase of the expression level of Tbet, GATA3 and RORg in comparison both with baseline and w/t group (Fig 6, compare C and B). The level of Tbet was 1.3 upregulated and the level of GATA3 was 2-fold upregulated in comparison with w/t group, data were statistically insignificant. The level of RORg was 5-fold upregulated (p = 0.019) in comparison with w/t group. Interestingly that the expression levels of these genes after second DC immunization did not differ from ones in w/t group (Fig 6C and 6B). The level of Foxp3 expression was downregulated similar to w/t group and prophylactic groups but data also were statistically insignificant.

## Discussion

In this work we studied prophylactic and therapeutic anti-tumor vaccines based on DCs loaded with tumor RNA or cell lysate. Three experimental murine tumor models characterized by the presence or absence of palpable primary tumor and metastases and generated from cell lines (B16) or by re-passaging of cells from primary tumor nodes were used. These murine tumor models have strong relevance to human tumors—namely, Krebs-2 adenocarcinoma (primary tumor), corresponds to human adenocarcinoma; B16 melanoma (metastatic tumor without a primary node), corresponds to widespread metastatic human melanoma and mimics a situation in which only metastases are clinically detectable, and Lewis lung carcinoma (LLC) (metastatic tumor with a primary node), corresponds to anaplastic human carcinoma.

In planning our study we expected to evaluate the advantage of one over the other scheme of vaccination. DC vaccination after tumor transplantation (Scheme 2) was the most effective in treating the solid Krebs-2 tumor (2-fold retardation of tumor growth in comparison with control) that corresponds to numerous data about the efficiency of therapeutic DC vaccines [19–23]. Prophylactic DC vaccines (Scheme 1) demonstrated high antimetastatic potential—the numbers of metastases were significantly decreased (4.7- and 10-fold) in the Lewis lung carcinoma and B16 melanoma models in comparison with the control.

The efficacy of therapeutic and prophylactic DC-based vaccines to inhibit primary tumor growth was demonstrated in a number of studies [7, 22, 29–31,33–36, 43, 44, 45]. However, there is little data about the efficacy of prophylactic DC-based vaccines in activation of antimetastatic response [37,38,39]. We show that prophylactic DC vaccines are mostly effective in inhibition of metastases but not primary tumor.

It should be mentioned that the efficacy of prophylactic and therapeutic DC-based vaccines primarily depends on tumor type. *In vivo* experiments showed that both prophylactic and therapeutic schemes of DC vaccination were efficient in tumor growth retardation in MC38/MUC1 colon cancer, LLC, EL4 thymoma and HPV16-associated MK16 tumor models [30, 31, 36, 37], whereas WEHI-164 fibrosarcoma and MC38/CEA colon cancer were more sensitive to prophylactic DC vaccines with low if any antitumor efficiency of therapeutic scheme of DC vaccination [34, 35]. The success of prophylactic DC vaccines can be explained by the absence of a tumor, which negatively affects DC functions and weakens cytotoxic T-lymphocyte and T helper anti-tumor responses [46, 47]. Prophylactic vaccination provides a well-timed activation of anti-tumor immune surveillance in the absence of tumor burden, the induction of specifically primed cytotoxic T-lymphocytes and has a positive effect on humoral immunity [48].

The success of the anti-tumor treatment with modified DCs depends also on the choice of an appropriate source of TAAs for DC loading. In our experiments we used autologous tumor cells for transplantation and tumor material for DC loading. Nevertheless we suppose that preventive treatment experiment can be extrapolated to clinical trial where allogeneic tumor material is used for DC-loading. We used sources of multiple antigens, tumor lysate and tumor-derived total RNA, as they contained a maximal assortment of antigens presented in tumor cells and their promotion could potentially launch broad polyclonal responses [49–51]. However, our data demonstrated that DCs loaded with tumor lysate were poorly immunogenic in the case of the Krebs-2 adenocarcinoma model: no inhibition of tumor growth was observed under either prophylactic (Scheme 1) or therapeutic (Scheme 2) conditions. Total tumor RNA as a source of TAA was the most effective: we observed a 1.9-fold decrease in tumor growth when a therapeutic DC vaccine loaded with Krebs-2 RNA was used.

MHC/peptide complexes are known to have a short half-life that limits the duration of antigen presentation [49]. In the case of lysate, antigen presentation lasts only one round until all of the proteins and peptides that entered into DCs have been processed and MHC/peptide complexes are assembled and presented on the cellular surface. RNA provides several rounds of TAA expression when delivered into DCs and hence a more efficient turnover rate of TAA/MHC I–II complexes between the cytosol and the cellular surface [52].

We expected that the efficiency of vaccination (anti-tumor/anti-metastatic response) would depend on the CD4^+^/CD8^+^ ratio and correlate with switching between Th1 and Th2 responses. The success of the therapeutic DC vaccine for Krebs-2 treatment accompanied by a drastic reduction in CD8^+^ cells in the spleens of mice on day 5 after Krebs-2 transplantation (one day after DC vaccination) can possibly be explained by recruiting of CD8^+^ cells by DCs in lymph nodes rather than suppression of the proliferation of CD8^+^ T-lymphocyte cells. In the case of the prophylactic DC-based vaccine we observed only activation of the CD4^+^ cell population and a slight decrease in the CD8^+^ component, that was not enough for efficient inhibition of tumor growth.

It is known that Th1/Th2 responses are in balance with each other. Th1 and Th2 cell differentiation is counter-regulatory and self-reinforcing; therefore, when one link is weakened the counter analogue is activated and vice versa [53]. We observed that significant metastasis suppression after single prophylactic DC vaccination in the B16 melanoma model was accompanied by the slight reductions in the levels of Th2-specific cytokines in the blood serum of experimental animals nevertheless the level of Th1/Th2/Th17 master regulators was not changed. Booster DC vaccination under prophylactic scheme that was not effective in metastases suppression was accompanied by two-fold statistically significant increase of Th17-master regulator RORg. Polarization of Th17-response associated with autoimmune and pro-inflammatory responses is a possible explanation of inefficiency of the booster prophylactic DC vaccination.

In the case of single therapeutic vaccination an increase of the expression levels of RORg and Tbet together with the increase of several Th1 specific cytokine content in bloodstream of mice was observed that is the evidence polarization of Th1/Th17 responses (see Fig 6C and S2 Fig).

To enhance the efficacy of anti-tumor treatment we used two DC vaccinations in the B16 melanoma model as it is commonly used worldwide. Surprisingly, our data show that booster DC vaccines failed to improve anti-tumor responses in either the prophylactic or therapeutic schemes of DC vaccination; nevertheless, inhibition of the number of metastases by a factor of 1.5–2 was observed but it was statistically insignificant. It partially agrees with the recently reported data where booster DC vaccination was detrimental in therapeutic settings of treatment of B16F1 murine melanoma [54]. Authors explain the absence of advantages to the anti-tumor effect of a booster DC vaccine by too-close booster DC injections that caused elimination of Ag-loaded DCs by effector cells in previously vaccinated animals, and hence reductions in the survival and functionality of central memory T cells [54]. Furthermore, in therapeutic settings of DC treatment cytotoxic T lymphocytes may be exhausted and/or tolerated due to excessive activation of tumour specific immunity by multiple exogenous boostings with DC vaccines and endogenous boosting with TAAs and pro-inflammatory factors released from dying tumor cells [54] that result in depletion of the immune system and weakening of the anti-tumor response.

DCs are actively studied as therapeutic cancer vaccines in various clinical trials [19–24]. However, the prophylactic potential of DC vaccines is beyond the scope of these investigations and requires further researches with taking into account histological type and malignancy level of tumors.

In our work it was demonstrated that prophylactic vaccines based on DCs loaded with tumor-derived RNA are promising in treatment of highly aggressive metastatic tumors and this prophylactic vaccine could be constituent part of efficient protocols targeted to metastasis inhibition.

## Supporting Information

S1 Fig Gating strategy of flow cytometry. CD8^+^ cell content in spleen of healthy mice C57Bl/6. Spleen cells were stained with anti-CD8-FITC mAbs. Control—non-stained spleen cells. Gating strategy of flow cytometry of CD4^+^ cells stained with PE-conjugated mAbs was similar to the above (in FL2 Log scale).
(PDF)

S2 Fig Box plots of Th1-specific cytokine content in the blood serum of mice with metastatic melanoma after prophylactic or therapeutic DC vaccination: (A) IL-12 (p40/p70), (B) IL-2, (C) IFN-γ. Boxes represent 25^th^, 50^th^, and 75^th^ percentiles. Squares with line represent median. Whiskers represent minimum/maximum. w/t—non-treated mice with metastatic melanoma injected with saline buffer. Type of DC vaccine is indicated as S-I/T/A where S—scheme of the treatment 1 or 2, I—immunization number, T—transfectant, A—antigen source. Data were statistically analysed using one-way ANOVA with post hoc Fisher test. *p* value indicates a statistically reliable difference.
(PDF)

S3 Fig Box plots of pro-inflammatory cytokine content in the blood serum of mice with metastatic melanoma after prophylactic or therapeutic DC vaccination: (A) IL-1β, (B) IL-6, (C) GM-CSF, (D) TNF-α. Boxes represent 25^th^, 50^th^, and 75^th^ percentiles. Squares with line represent median. Whiskers represent minimum/maximum. w/t—non-treated mice with metastatic melanoma injected with saline buffer. Type of DC vaccine is indicated as S-I/T/A where S—scheme of the treatment 1 or 2, I—immunization number, T—transfectant, A—antigen source. Data were statistically analysed using one-way ANOVA with post hoc Fisher test. *p* value indicates a statistically reliable difference.
(PDF)
