# Supplementary material for: Prophylactic Dendritic Cell-Based Vaccines Efficiently Inhibit Metastases in Murine Metastatic Melanoma
Source: PLoS One. 2015 Sep 1;10(9):e0136911. doi: 10.1371/journal.pone.0136911 (PMC4556596; doi:10.1371/journal.pone.0136911)
Supplement: S1 Fig — Spleen cells were stained with anti-CD8-FITC mAbs. Control—non-stained spleen cells. Gating strategy of flow cytometry of CD4+ cells stained with PE-conjugated mAbs was similar to the above (in FL2 Log scale). (PDF) [file pone.0136911.s001.pdf]

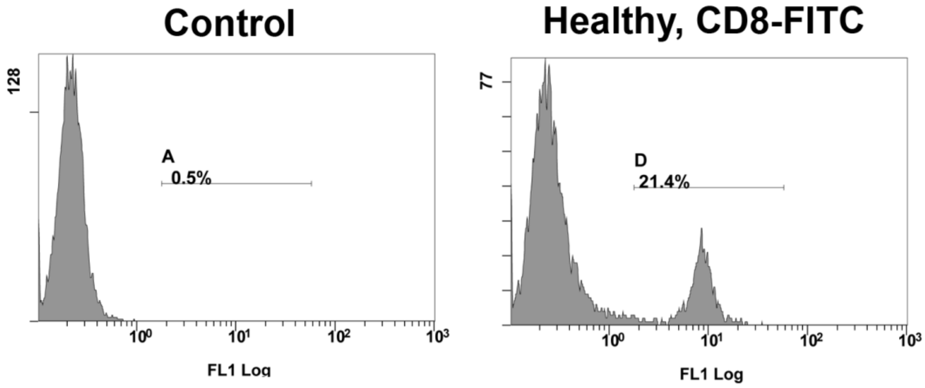

S1 Fig. Gating strategy of flow cytometry. CD8$^+$ cell content in spleen of healthy mice C57Bl/6. Spleen cells were stained with anti-CD8-FITC mAbs. Control – non-stained spleen cells. Gating strategy of flow cytometry of CD4$^+$ cells stained with PE-conjugated mAbs was similar to the above (in FL2 Log scale).
