# Supplementary material for: Prophylactic Dendritic Cell-Based Vaccines Efficiently Inhibit Metastases in Murine Metastatic Melanoma
Source: PLoS One. 2015 Sep 1;10(9):e0136911. doi: 10.1371/journal.pone.0136911 (PMC4556596; doi:10.1371/journal.pone.0136911)
Supplement: S2 Fig — Boxes represent 25th, 50th, and 75th percentiles. Squares with line represent median. Whiskers represent minimum/maximum. w/t—non-treated mice with metastatic melanoma injected with saline buffer. Type of DC vaccine is indicated as S-I/T/A where S—scheme of the treatment 1 or 2, I—immunization number, T—transfectant, A—antigen source. Data were statistically analysed using one-way ANOVA with post hoc Fisher test. p value indicates a statistically reliable difference. (PDF) [file pone.0136911.s002.pdf]

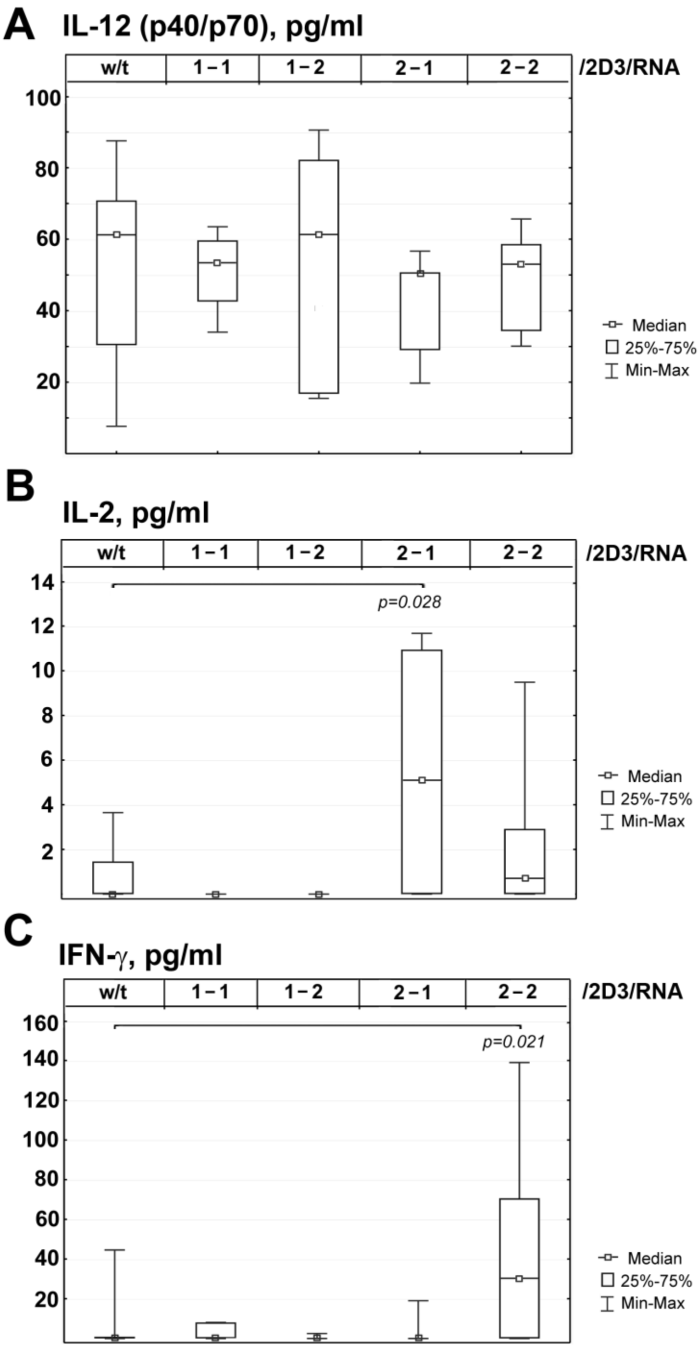

S2 Fig. Box plots of Th1-specific cytokine content in the blood serum of mice with metastatic melanoma after prophylactic or therapeutic DC vaccination: (A) IL-12 (p40/p70), (B) IL-2, (C) IFN-γ. Boxes represent 25th, 50th, and 75th percentiles. Squares with line represent median. Whiskers represent minimum/maximum. w/t – non-treated mice with metastatic melanoma injected with saline buffer. Type of DC vaccine is indicated as S-I/T/A where S – scheme of the treatment 1 or 2, I - immunization number, T – transfectant, A – antigen source. Data were statistically analysed using one-way ANOVA with post hoc Fisher test. *p* value indicates a statistically reliable difference.
